# 

**Published:** 1895-11

**Authors:** 


					﻿Vol. XV.
NOVEMBER, 1895.
NO. 11.
THE
Homeopathic pHY^iCiAfl,
A Monthly Journal of Homoeopathic Materia Medica
and Clinical Medicine.
** He is a freeman whom truth makes free, and all are slaves beside."
"Seek the truth: come whence it may, cost what it will."
BDITBD AND PDBLMHED BT
WALTER M. JAMES, M. E>.
PHILADELPHIA:
USTo. 1231 LOCUST STREET.
XCO Q IT :
ALFRED HEATH! & CO.,r8OLB! Agents .'fob Gbeat Bbitain,
114) Ebury Street, Eaton Square.
9^ Yearly Subscription, $2.50, invariably in advance. Single numbers, 95 etc.
Entered at the Philadelphia Poit-Offlce at Second-Claw Mall Matter.
				

